# Selenium-Based Strategies for Targeting Multidrug-Resistant Breast Cancer: A Review

**DOI:** 10.3390/ijms27093848

**Published:** 2026-04-26

**Authors:** Hubert Bajer, Klementyna Kupisz, Szymon Jóźwiak, Angelika Długosz-Pokorska

**Affiliations:** Department of Biomolecular Chemistry, Faculty of Medicine, Medical University of Lodz, 92-215 Lodz, Poland; hubert.bajer@student.umed.lodz.pl (H.B.); klementyna.kupisz@student.umed.lodz.pl (K.K.); szymon.jozwiak@student.umed.lodz.pl (S.J.)

**Keywords:** selenium compound, breast cancer, paclitaxel, doxorubicin, tamoxifen, trastuzumab

## Abstract

Breast cancer remains a major global health challenge, necessitating the development of effective anticancer strategies to overcome drug resistance and reduce the adverse effects of chemotherapy. Selenium-based therapies have demonstrated promising anticancer activity in various experimental models, including drug-resistant breast cancer cells. Selenium is an essential micronutrient required for the proper functioning of numerous biological processes in human cells. Selenoproteins play key roles in antioxidant defense, redox regulation, and immune system function. Selenium-containing compounds are characterized by high specificity, relatively low toxicity, and favorable cell membrane permeability, which supports their potential application in precision medicine. These compounds can inhibit cancer cell growth through multiple mechanisms, including modulation of redox balance, induction of apoptosis, and interference with signaling pathways involved in tumor progression. This review summarizes current knowledge on the mechanisms by which selenium compounds affect drug-resistant breast cancer cells, highlights key experimental findings, and discusses their potential use as adjuncts to conventional therapies.

## 1. Introduction

Breast cancer remains a major global health challenge and a leading cause of morbidity and mortality worldwide. Despite substantial progress in chemotherapy, disease control is still limited by tumor heterogeneity, the development of therapeutic resistance, and a high risk of relapse [1,2]. Conventional treatment strategies include surgery, chemotherapy and radiotherapy. These approaches are often associated with significant toxicity and limited specificity toward cancer cells. The emergence of drug resistance further reduces treatment efficacy and remains a major challenge in breast cancer treatment. In addition, standard therapies frequently fail to prevent metastatic progression and recurrence. These limitations highlight the need for more effective and targeted therapeutic strategies [3,4,5].

Selenium is an essential micronutrient required for many biological processes in human cells [6,7]. It is a component of selenoproteins involved in antioxidant defense, redox regulation, metabolism and immune function. Adequate selenium levels are necessary to maintain cellular homeostasis and to protect cells from oxidative stress–related damage [8]. Interest in selenium compounds as potential anticancer agents has increased in recent years. Experimental studies show that these compounds can inhibit cancer cell growth through several mechanisms. These include the induction of apoptosis and the modulation of redox balance. Selenium compounds also affect signaling pathways involved in tumor progression. These findings have supported further research on selenium-based therapeutic strategies [8].

This study aims to summarize and critically evaluate experimental evidence on selenium compounds in drug-resistant breast cancer models. The analysis is based on preclinical studies published between 2005 and 2026, identified through PubMed, Scopus, and Web of Science. Despite the large number of studies, the translational potential of selenium compounds remains unclear. Most data come from in vitro studies, while in vivo validation is limited and clinical evidence is lacking. The available studies show substantial heterogeneity in experimental design, types of selenium compounds, dosing strategies, and model systems. In many cases, statistical reporting is incomplete, and effect sizes are not consistently provided. This limits direct comparison between studies. The predominance of small-scale preclinical research, together with potential publication bias, further weakens the overall strength of the evidence. Overall, selenium compounds represent a biologically active and well-studied group of agents. However, their clinical application in breast cancer therapy remains insufficiently developed. This review integrates current preclinical findings and critically assesses their translational limitations in drug-resistant breast cancer. The analysis includes both natural and synthetic selenium compounds tested in models resistant to trastuzumab, doxorubicin, tamoxifen, and paclitaxel. Particular attention is given to their mechanisms of action and their potential use as adjuncts to established therapies [1,2,3,4,5,6,7,8,9,10,11,12,13,14,15,16,17,18,19,20,21,22,23,24,25,26,27,28,29,30,31,32,33,34,35,36,37,38,39,40,41,42,43,44,45,46,47,48,49,50,51,52,53,54,55,56,57,58,59,60,61,62,63,64,65,66,67,68,69,70,71,72,73,74,75,76,77,78,79,80,81,82,83,84,85,86,87,88,89,90].

## 2. Biological Role of Selenium

Selenium metabolism in humans begins with the dietary intake of both organic (selenomethionine, selenocysteine) and inorganic (selenate, selenite) forms. These compounds are primarily absorbed in the small intestine. Organic selenium, particularly selenomethionine, is transported via amino acid transport systems and can be nonspecifically incorporated into proteins in place of methionine, thereby serving as a storage form of selenium. In contrast, inorganic selenium is absorbed through both active and passive transport mechanisms and undergoes intracellular reduction [8].

Regardless of its initial chemical form, selenium is ultimately metabolized to selenide (H_2_Se), which represents the central intermediate in selenium metabolism. Organic forms are converted into selenocysteine and subsequently degraded to selenide, whereas inorganic forms such as selenite are reduced via glutathione- and thioredoxin-dependent pathways [8].

Selenide is utilized for the biosynthesis of selenocysteine, which is co-translationally incorporated into selenoproteins through a highly specific mechanism involving selenocysteine-specific tRNA and SECIS elements in mRNA. Selenoproteins constitute a distinct class of proteins characterized by the presence of selenocysteine, the 21st amino acid, inserted at specific UGA codons. These proteins play essential roles in maintaining cellular homeostasis, primarily through redox regulation and antioxidant defense [7,9].

Key selenoproteins include glutathione peroxidases, thioredoxin reductases, and iodothyronine deiodinases. Glutathione peroxidases reduce reactive oxygen species, whereas thioredoxin reductases regulate redox-sensitive signaling pathways. Iodothyronine deiodinases are critical for thyroid hormone metabolism. In addition, selenoproteins contribute to immune regulation, protein folding, and protection against oxidative stress–related damage, making them important mediators of cellular function and potential targets in cancer therapy [10,11,12].

Excess selenium is detoxified through methylation reactions, leading to the formation of methylated metabolites such as dimethylselenide and trimethylselenonium, which are excreted via the lungs and urine. Selenium stored as selenomethionine within proteins can be gradually released during normal protein turnover, thereby contributing to the maintenance of selenium homeostasis (Table 1).

**Figure 1:**
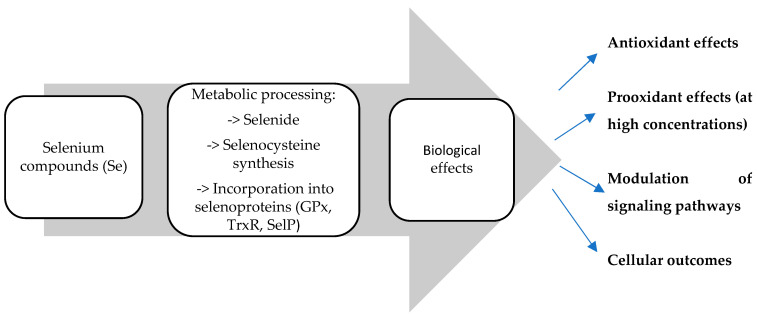
Schematic representation of the mechanisms of action of selenium compounds in the human body. The figure is an original illustration created by the authors based on data and concepts derived from the literature cited in this review [7,8,9,10,11,12,13,14,15,16,17,18].

In addition, the dual redox nature of selenium allows it to function as a pro-oxidant at higher concentrations, selectively inducing cytotoxicity in cancer cells while sparing normal cells [7,8,9,10,11,12,13,14,15,16,17,18] (Figure 1).

## 3. Selenium Compounds: Classification and Characteristics

### 3.1. Classification of Selenium Compounds

Selenium is a chemical element that exists in various forms ranging from elemental selenium (Se^0^) to inorganic, organic, and synthetic selenium compounds [19]. Selenium compounds are generally classified into inorganic, organic, and synthetic compounds, each with distinct chemical characteristics and applications [20]. The inorganic forms are elementary selenide (Se^2−^), selenate (SeO_4_^2−^) and selenite (SeO_3_^2−^). Organic selenium forms, which are essential for living organisms, include the most important ones, which are selenocysteine, selenomethionine, methylselenocysteine and proteins, which have those amino acids [21]. The last class to be distinguished is synthetic selenium forms, where focus shifts towards their low toxicity, anticancer properties and possibilities as diagnostic tools, mostly in the form of selenium nanoparticles (SeNPs) [22]. Despite their different forms, these selenium groups can be used for biomedical purposes, contributing to human health benefits.

### 3.2. Inorganic Selenium Compounds

Inorganic selenium compounds primarily include sodium selenite (Na_2_SeO_3_), sodium selenate (Na_2_SeO_4_), and selenium dioxide (SeO_2_). These compounds have been studied for their potential anticancer activity as well as their use in nutritional supplementation [23].

Sodium selenite and sodium selenate are water-soluble selenium salts commonly used in dietary and experimental supplementation. Selenite is generally more reactive than selenate due to its lower oxidation state [6]. Both compounds are absorbed in the small intestine, although via different transport mechanisms. Selenate is actively transported via sodium-dependent transporters, whereas selenite is mainly absorbed through passive diffusion [7]. Following absorption, both forms are metabolized to hydrogen selenide (H_2_Se), which represents a key intermediate in selenium metabolism [24]. Selenide is subsequently used for the biosynthesis of selenocysteine and the production of selenoproteins, including glutathione peroxidases and thioredoxin reductases, which are essential for cellular redox regulation [19].

At higher concentrations, hydrogen selenide can contribute to oxidative stress through the generation of reactive oxygen species, leading to DNA damage and cellular toxicity [19]. In contrast, at lower concentrations, inorganic selenium compounds may exert therapeutic effects, particularly through modulation of redox balance.

Sodium selenite has been shown to induce cytotoxicity in experimental models, including fish, through oxidative stress–mediated activation of caspase-3/7 and subsequent apoptosis [25]. Selenium accumulation from dietary sources such as mushrooms is also relevant, as these organisms can retain water-soluble selenium forms that may be further converted into organic selenium compounds in biological systems [27]. Sodium selenate has additionally been investigated in animal models of neurodegeneration. In a rat model of traumatic brain injury, selenate administration reduced levels of phosphorylated tau protein, which was associated with improved cognitive function and reduced brain atrophy [25,26,27,28]. Overall, inorganic selenium compounds exhibit a dual biological role, acting as both potential cytotoxic agents at higher concentrations and modulators of cellular function at lower doses, which supports their interest in both therapeutic and nutritional contexts.

### 3.3. Organic Selenium Compounds

Organic selenium compounds contain selenium covalently bonded to carbon atoms. This structure is associated with lower toxicity and greater bioavailability than inorganic selenium forms [29]. The most important groups include selenoamino acids, such as selenocysteine and selenomethionine, as well as methylselenol and its precursors [30]. Selenocysteine is the 21st amino acid encoded by the UGA codon, which is a stop codon. Incorporation into proteins requires a specific selenocysteine tRNA [31]. Selenocysteine incorporation is essential because it depends on the *Trsp* gene encoding the specific tRNA. The deletion of this gene results in oxidative stress and further embryonic death in mice. Moreover, the knockout of this gene also increased susceptibility to malignant tumors [31]. Selenomethionine is a naturally occurring amino acid in which a selenium atom replaces sulfur. Selenomethionine is nonspecifically incorporated in place of methionine [30]. The function of this process is to produce other selenium compounds, as well as to be the storage site to produce selenoproteins. Selenoproteins exhibited peroxidase activity due to the presence of selenium, get peroxidase activity, lower than that of classical peroxidases but greater than compounds with sulfuric atom [32,33].

Another subgroup of organic selenium compounds is methylselenol and its derivatives. Methylselenol (CH_3_SeH) is the metabolite of selenoamino acids such as selenomethionine or selenocysteine, which is generated through metabolic conversion involving the Se-methyl group [34]. This modification offers better anticancer protection through halting angiogenesis. Methylselenol induced apoptosis and caused cell cycle arrest in melanoma cells, which is a promising effect for future therapeutic advantages [35,36,37,38,39]. Precursors of methylselenol also enhance MHC class I expression, which prevents tumors from escaping cytotoxic T lymphocytes [36]. This supports chemoprotective activity, much needed when dealing with cancer. Methylselenol also induces the expression of the NKG2D ligands, which are only expressed in damaged or infected cells. This can be used for improving that expression in cancer cells, which would result in leading to their destruction by NKG2D-expressing effector cells [36,37,38,39].

### 3.4. Synthetic Selenium Compounds

Synthetic selenium compounds represent a diverse group of selenium-containing molecules that exhibit properties of therapeutic interest, including enzyme-mimetic activity, anticancer potential, and antioxidant effects, which are key features of selenium-based compounds. Within this group, recent research has focused on selenides and diselenides. Selenides (R–Se–R′) are compounds in which selenium atoms are covalently bonded to carbon atoms [40,41,42,43,44,45].

Synthetic selenium compounds have a wide range of therapeutic applications, including redox activity, anticancer effects, antimicrobial properties, and functional similarity to selenoproteins. Ebselen, a heterocyclic glutathione peroxidase mimetic, has demonstrated antifungal activity, with an inhibitory rate of 90% at a concentration of 10 μg/mL [46]. In addition, ebselen induces oxidative stress in fungal cells, which may be exploited as a therapeutic strategy in fungal infections [46]. Ebselen has also shown inhibitory activity against SARS-CoV-2 by targeting the main protease, an enzyme essential for viral replication [47].

Bis(2-arylimidazo[1,2-a]pyridin-3-yl) diselenides also exhibit significant biological activity, including strong cytotoxic effects against cancer cells, resulting in markedly reduced viability of HeLa cells while maintaining low toxicity toward non-cancerous cells [48]. Both diselenides and selenides reduce cancer cell viability and inhibit glutathione S-transferases, which are often overexpressed in cancer cell lines such as HeLa and MCF-7 [49]. In addition, diselenides have been investigated as selective insecticides, as they can induce mortality in Aedes aegypti larvae [50].

Recently, scientific interest has shifted toward selenium nanoparticles (SeNPs). These nanostructures exhibit properties similar to selenium ions but with significantly lower toxicity to normal cells, making them promising candidates for therapeutic applications. SeNPs can act as antibody adjuvants in the treatment of bacterial infections such as those caused by Staphylococcus aureus, enhancing cellular immune responses [51]. Furthermore, selenium nanorods have demonstrated anticancer activity against MCF-7 and Hep-G2 cell lines, reducing their viability in treated cultures [52].

SeNPs can also function as drug delivery systems due to their ability to undergo conjugation, electrostatic interactions, and encapsulation of therapeutic agents, thereby improving therapeutic efficacy [53]. When combined with polysaccharides, SeNPs have been shown to inhibit tumor growth through apoptosis, as confirmed by DNA fragmentation assays [54]. In addition, SeNPs have been explored in nutritional applications, where they increase selenium content in plants such as soybean, offering a potential strategy for addressing selenium deficiency [55].

Overall, these examples demonstrate the broad range of applications of synthetic selenium compounds and highlight their significant potential for further biomedical development.

## 4. Mechanisms of Resistance in Breast Cancer

Breast cancer remains one of the most common malignancies in women worldwide. MDR in breast cancer therapy represents a growing clinical challenge. Both intrinsic and acquired resistance mechanisms enable cancer cells to evade cytotoxic effects [4].

One of the most important mechanisms involves alterations in drug transport, including overexpression of ABC transporters such as ABCB1 and ABCG2, which reduce intracellular drug accumulation. Dysregulation of apoptotic pathways, including altered expression of Bcl-2, Bax, and cytochrome c, inhibits caspase-mediated cell death. In addition, enhanced antioxidant systems such as glutathione, thioredoxin, and selenoproteins buffer reactive oxygen species (ROS), maintain redox homeostasis, and reduce drug-induced cytotoxicity.

In resistant cells, pro-survival signaling pathways, including PI3K/Akt/mTOR, NF-κB, MAPK/ERK, and Src, are frequently dysregulated, leading to increased proliferation, enhanced survival, and reduced sensitivity to apoptosis. The MDR phenotype in breast cancer is also associated with epigenetic alterations, including DNA methylation, histone modifications, and regulation by microRNAs and long non-coding RNAs, which modulate genes involved in DNA repair, apoptosis, and drug metabolism [3,4,5].

Another important mechanism involves cancer stem cells, which contribute to therapeutic resistance through slow cell cycle progression, enhanced DNA repair capacity, and increased drug efflux activity. Tumor heterogeneity and microenvironmental factors, including hypoxia, stromal-derived cytokines, and extracellular matrix interactions, provide additional protection against chemotherapy. Collectively, these mechanisms highlight the multifactorial nature of drug resistance in breast cancer and underscore the need for novel therapeutic strategies [3,4,5]. These mechanisms are directly relevant to the effects of selenium compounds discussed in this review. Dysregulated apoptotic pathways, including altered Bcl-2/Bax balance, may be targeted by selenium compounds that induce apoptosis through caspase activation and mitochondrial dysfunction. Enhanced antioxidant defenses in resistant cells, including glutathione and thioredoxin systems, may be disrupted by selenium-induced redox imbalance, leading to increased ROS levels and selective cytotoxicity in cancer cells.

**Figure 2:**
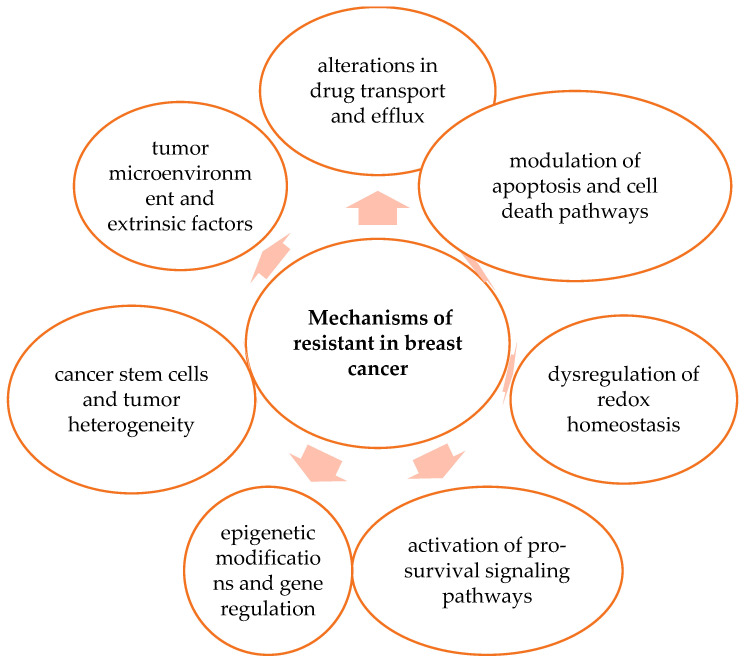
Mechanisms of resistance in breast cancer. Schematic overview of the main molecular and cellular pathways involved in therapeutic resistance. The figure is an original illustration created by the authors based on data and concepts derived from the literature cited in this review [3,4,5,6,7,8,9,10,11,12,13,14,15,16,17,18,19,20,21,22,23,24,25].

Moreover, selenium compounds have been shown to inhibit key signaling pathways such as PI3K/Akt, NF-κB, and MAPK, which are frequently upregulated in multidrug-resistant breast cancer and support cancer cell survival. Together, these findings suggest that selenium compounds may represent promising candidates for overcoming MDR in breast cancer. Novel anticancer agents, including selenium compounds and selenium nanoparticles, may overcome resistance by simultaneously modulating ROS balance, apoptotic signaling, and major survival pathways, thereby potentially improving sensitivity to conventional chemotherapy [3,4,5,6,12,19,25]. Figure 2 presents a schematic overview of the main molecular and cellular pathways involved in therapeutic resistance.

## 5. Mechanisms of Selenium Action in Resistant Breast Cancer Cells

Selenium compounds have attracted significant attention as potential therapeutic agents in resistant breast cancer due to their diverse biological activities and ability to target multiple pathways involved in tumor progression and resistance. Studies have shown that selenium compounds can produce significant anticancer effects while displaying lower toxicity toward normal tissues, making them promising candidates for selective therapy [56].

The anticancer activity of selenium compounds is mediated through several interconnected molecular mechanisms that disrupt essential processes necessary for cancer cell survival and proliferation. These mechanisms include the induction of apoptosis, modulation of cellular redox balance and ROS production, cell cycle arrest, and inhibition of tumor cell proliferation. Selenium derivatives may also influence key cell survival pathways and enhance the efficacy of conventional anticancer drugs when used in combination therapy [56,57,58,59,60,61,62,63,64,65,66].

Importantly, the anticancer effects of selenium compounds may vary depending on the molecular subtype of breast cancer. In luminal (ER-positive) models, such as MCF-7 cells, selenium nanoparticles have been shown to induce apoptosis primarily through oxidative stress and mitochondrial dysfunction, accompanied by modulation of Bcl-2 family proteins and caspase activation [57]. In contrast, in HER2-positive and therapy-resistant breast cancers, selenium exerts its effects through the inhibition of key survival pathways, particularly the PI3K/Akt signaling, thereby reducing proliferation and enhancing sensitivity to targeted therapies such as trastuzumab [66]. Notably, selenium compounds demonstrate strong anticancer potential in triple-negative breast cancer (TNBC), a highly aggressive subtype lacking effective targeted therapies. In TNBC models, selenium-based agents and nanoparticles have been reported to induce ROS-mediated apoptosis, cell cycle arrest, and inhibition of metastatic behavior, highlighting their potential as therapeutic adjuvants in this difficult-to-treat subtype [55,56,57,58,59,60,61,62,63,64,65].

Selenium compounds have also been shown to interact synergistically with various chemotherapeutic agents, potentially overcoming multidrug resistance by affecting mechanisms such as drug efflux and oxidative stress regulation. These properties make selenium compounds promising candidates for improving treatment outcomes in resistant cancers [56].

The following sections discuss the principal molecular mechanisms underlying the anticancer activity of selenium compounds, including apoptosis induction, redox modulation and ROS generation, cell cycle regulation, modulation of survival signaling pathways, and their synergistic interactions with conventional anticancer therapies [56,57,58,59,60,61,62,63,64,65,66].

## 6. Induction of Apoptosis

One of the primary mechanisms through which selenium compounds exert anticancer activity is the induction of apoptosis, or programmed cell death. Selenium has demonstrated significant anticancer potential in a wide range of malignancies, including breast, prostate, lung cancers, as well as melanoma and glioma. In recent years, SeNPs have gained attention due to their high biocompatibility, relatively low toxicity, and diverse biological activities, including the ability to induce apoptosis in cancer cells [57].

Apigenin (4′,5,7-trihydroxyflavone) is a naturally occurring flavone primarily found in plants of the Asteraceae family. It exhibits a broad spectrum of biological activities, including antioxidant, anti-inflammatory and anticancer properties. Importantly, apigenin has been shown to induce apoptosis and cell cycle arrest in cancer cells. Previous studies have shown that it can target both estrogen receptor-α (ERα)-dependent and ERα-independent pathways in breast cancer cells and regulate apoptotic signaling pathways involving p53 [57].

The apoptotic potential of selenium-based nanomaterials has been shown in breast cancer models. For example, biosynthesized selenium nanoparticles conjugated with apigenin (SeNPs-apigenin) were investigated for their anticancer effects in MCF-7 breast cancer cells. These nanoparticles significantly reduced cell viability and induced apoptosis in a dose-dependent manner. The compound inhibited the growth of MCF-7 cells with an IC_50_ value of 51.74 µM, although its cytotoxic activity was lower than that of doxorubicin (DOX), which showed an IC_50_ value of 0.59 µM in the same experimental model [57].

Further molecular analysis revealed that treatment with SeNPs-apigenin significantly increased apoptotic cell death compared with untreated control cells. This effect was associated with downregulation of Bcl-2 and a decreased Bcl-2/Bax ratio, together with upregulation of Bax and caspase-3. These findings indicate activation of intrinsic apoptotic signaling pathways [57].

In addition to modulating apoptotic regulators, SeNPs-apigenin treatment was found to promote oxidative stress, characterized by increased levels of ROS, nitric oxide (NO), and malondialdehyde (MDA), along with decreased levels of antioxidant markers such as glutathione (GSH), superoxide dismutase (SOD), and GPx. Elevated ROS levels can disrupt mitochondrial integrity and trigger the release of pro-apoptotic factors, leading to activation of caspase cascades and apoptotic cell death. These findings highlight the important role of oxidative stress in selenium-induced apoptosis in breast cancer cells [57].

### 6.1. Redox Modulation and Reactive Oxygen Species Generation

Selenium plays a dual role in redox biology, acting as both an antioxidant and a pro-oxidant, depending on its chemical form, concentration, and cellular environment. At physiological concentrations, selenium exhibits redox-active properties and contributes to the maintenance of cellular redox homeostasis. However, at higher concentrations, selenium may become genotoxic and potentially carcinogenic, primarily due to excessive production of ROS and disruption of cellular redox balance. To exert its antioxidant and anticancer activities, selenium must be incorporated into the structure of selenoproteins. To date, 25 selenoproteins have been identified in humans, all of which contain the amino acid selenocysteine in their active site. Several of these proteins exhibit significant redox activity, including thioredoxin reductases (TrxRs), GPxs, selenoprotein P (SelP), F (SelF), S (SelS), and M (SelM), which play key roles in supporting intracellular redox balance and protecting cells from oxidative damage [58].

One of the best examples of selenium’s protective role against oxidative stress is its involvement in the intracellular glutathione redox system (GSH/GSSG). In this system, reduced glutathione (GSH) acts as a major cellular antioxidant by neutralizing free radicals and reactive oxygen species through the catalytic activity of glutathione peroxidases. During this process, GPx enzymes oxidize GSH to glutathione disulfide (GSSG). The oxidized form is subsequently reduced back to GSH by NADPH-dependent glutathione reductase, thereby maintaining cellular redox homeostasis. Several GPx isoforms are selenium-dependent enzymes, highlighting the crucial role of selenium in protecting cells against oxidative stress and maintaining proper antioxidant defenses [58].

However, when selenium is present in excessive amounts, its effects on cellular redox status may become detrimental. Certain selenium compounds can participate in thiol oxidation and ROS generation, leading to disruption of redox homeostasis and oxidative damage to cellular components. Elevated ROS levels may cause damage to DNA, proteins, and lipids, ultimately impairing normal cellular functions [58].

Interestingly, this pro-oxidant property of selenium can be exploited in anticancer therapy. Many selenium compounds have been shown to induce ROS generation in cancer cells, including drug-resistant breast cancer cells, thereby promoting oxidative stress. Because cancer cells exhibit higher basal levels of oxidative stress than normal cells, they are more vulnerable to further increases in ROS levels. Selenium-induced ROS accumulation can therefore selectively damage cancer cells, triggering oxidative stress-mediated apoptosis, and inhibiting tumor growth [59].

Furthermore, selenium compounds may disrupt cellular redox balance by interacting with thiol-containing molecules such as glutathione and by modulating the activity of redox-regulating enzymes. This selective redox modulation contributes to the preferential cytotoxicity of selenium compounds toward cancer cells, while normal cells equipped with more efficient antioxidant defense systems are generally less affected [59].

### 6.2. Cell Cycle Arrest and Inhibition of Proliferation

Another important mechanism underlying the anticancer activity of selenium compounds is the inhibition of cell cycle progression. Selenium-based agents have been shown to induce cell cycle arrest at critical regulatory checkpoints, particularly at the G0/G1 and G2/M phases, thereby preventing uncontrolled proliferation of cancer cells. These effects are mediated through the modulation of key regulators of the cell cycle, including cyclins, cyclin-dependent kinases (CDKs), and CDK inhibitors such as p21 and p27, which collectively regulate progression through the cell cycle. By disrupting these regulatory pathways, selenium compounds can halt cell division and suppress tumor growth in breast cancer cells, including those showing chemoresistance or hormonal resistance [60,61].

Recent studies have demonstrated that selenium nanoparticles can effectively inhibit the proliferation of breast cancer cells by inducing G2/M phase arrest, which represents a critical checkpoint responsible for ensuring proper DNA replication and genomic integrity before mitosis. In breast cancer models, biosynthesized SeNPs have been shown to interfere with cell cycle regulatory mechanisms, resulting in G2/M phase accumulation and suppression of tumor cell growth. This effect is associated with alterations in the expression of proteins regulating cell cycle progression and mitosis, ultimately preventing cancer cells from completing mitosis and generating daughter cells [60].

Similarly, selenium speciation influences its ability to regulate cell cycle dynamics. Certain selenium compounds can enhance G2/M checkpoint activation, which not only inhibits cell proliferation but also sensitizes cancer cells to therapeutic interventions such as radiotherapy. The induction of G2/M arrest increases cellular vulnerability to DNA damage, as cells at this stage of the cycle are particularly sensitive to stress and genomic instability. As a result, selenium-induced cell cycle disruption can potentiate the effectiveness of anticancer treatments while simultaneously suppressing tumor cell growth [61].

Importantly, cell cycle arrest induced by selenium compounds often occurs in parallel with apoptotic signaling pathways. The prolonged inhibition of cell cycle progression allows for the accumulation of cellular stress, DNA damage, and oxidative imbalance, which can ultimately trigger programmed cell death. This dual mechanism—simultaneous inhibition of proliferation and induction of apoptosis—is particularly advantageous in resistant cancer cells that have developed mechanisms to evade conventional cytotoxic therapies. Consequently, selenium compounds are promising candidates for targeting multiple vulnerabilities in breast cancer cells and enhancing anticancer efficacy [61].

### 6.3. Modulation of Survival Signaling Pathways

Selenium compounds can also exert anticancer effects through the modulation of intracellular signaling pathways that regulate cell survival, proliferation, and therapy resistance. Several signaling cascades are frequently dysregulated in cancer cells, including the phosphoinositide-3-kinase/protein kinase B (PI3K/Akt), the mitogen-activated protein kinase (MAPK), and NF-κB pathways, all of which play central roles in tumor development and therapy resistance. Dysregulation of these pathways contributes to enhanced cell proliferation, inhibition of apoptosis, and increased metastatic potential in many cancers, including resistant breast cancer [62].

One of the most important signaling networks involved in cancer progression is the PI3K/Akt pathway, which regulates multiple cellular processes such as cell growth, metabolism, survival, and proliferation. Hyperactivation of this pathway is commonly observed in cancer and is strongly associated with drug resistance and poor prognosis. Selenium compounds have been shown to interfere with PI3K/Akt signaling, leading to reduced activation of downstream targets that promote cell survival. Inhibition of this pathway decreases proliferative signaling and promotes apoptotic responses in cancer cells, suppressing tumor growth and increasing sensitivity to anticancer therapies [62].

Selenium may also influence the MAPK signaling pathway, which plays a critical role in controlling cellular responses to stress and growth signals. The MAPK pathway includes several kinase cascades, such as ERK, JNK, and p38, each of which regulates different cellular processes. Selenium compounds have been shown to suppress ERK signaling, which is typically associated with cell proliferation and survival, while simultaneously activating stress-related kinases such as JNK and p38. Activation of these stress-responsive kinases promotes apoptotic signaling and contributes to the elimination of cancer cells [63].

Another important molecular target of selenium compounds is the NF-κB signaling pathway, a transcription factor complex that regulates the expression of genes involved in inflammation, cell survival, angiogenesis, and resistance to therapy. Persistent activation of NF-κB is commonly observed in cancer cells and contributes to the expression of anti-apoptotic and pro-survival proteins. Selenium compounds have been shown to suppress NF-κB activation, thereby reducing the expression of genes that promote tumor cell survival and resistance to treatment. This inhibition enhances apoptotic responses and sensitizes cancer cells to anticancer therapies [63].

**Figure 3:**
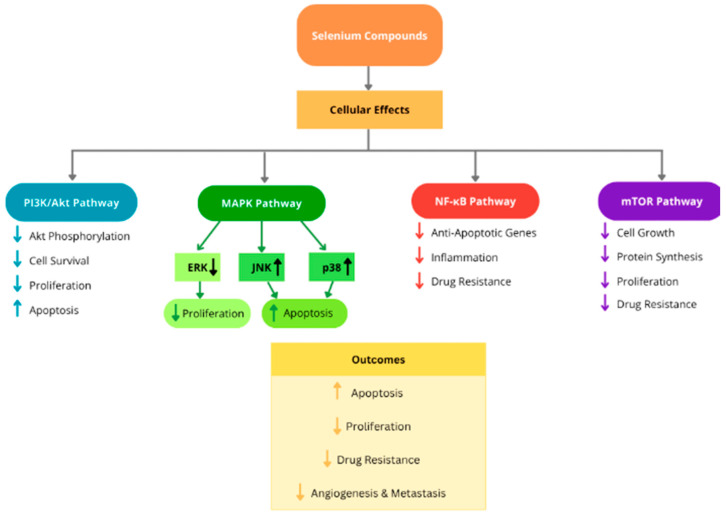
Schematic representation of the effects of selenium compounds on major signalling pathways in cancer cells. Selenium inhibits pro-survival pathways such as PI3K/Akt, NF-κB, and mTOR, while modulating MAPK signalling by suppressing ERK and activating stress-related kinases (JNK and p38). These combined effects result in increased apoptosis, reduced proliferation, and decreased drug resistance. The figure is an original illustration created by the authors based on data and concepts derived from the literature cited in this review [57,58,59,60,61,62,63,64].

Evidence from experimental studies further supports the role of selenium in modulating these signaling networks. In an in vivo study, selenium nanoparticles were shown to enhance the therapeutic efficacy of sorafenib, a multikinase inhibitor used in cancer treatment. Selenium nanoparticles reduced drug resistance in liver cancer cells by modulating mTOR and NF-κB signaling pathways, while also contributing to decreased angiogenesis and metastasis. These findings highlight the ability of selenium compounds to influence multiple molecular pathways simultaneously, thereby improving therapeutic responses and overcoming resistance mechanisms in cancer cells [64]. Figure 3 summarizes the effects of selenium compounds on key intracellular signaling pathways involved in cancer cell survival, proliferation, and therapy resistance.

### 6.4. Synergy with Conventional Therapies

An important advantage of selenium compounds is their ability to enhance the effectiveness of conventional breast cancer therapies. Several studies have demonstrated that selenium can sensitize resistant breast cancer cells to commonly used chemotherapeutic and targeted agents, including doxorubicin, paclitaxel, trastuzumab, and tamoxifen. Through these interactions, selenium compounds may improve therapeutic responses and overcome drug resistance that frequently limit the efficacy of standard anticancer treatments [65,66,67].

One example of this synergistic interaction was demonstrated in a study investigating the effects of selenium on MCF-7 breast cancer cells treated with doxorubicin. Selenium supplementation increased the sensitivity of cancer cells to doxorubicin-induced apoptosis by modulating the Akt signaling pathway and its downstream substrates, which are important regulators of cell survival. Inhibition of Akt phosphorylation reduced pro-survival signaling and promoted apoptotic cell death, thereby enhancing the cytotoxic activity of doxorubicin [65].

Similarly, selenium has been shown to inhibit the growth of trastuzumab-resistant breast cancer cells. In these cells, selenium treatment reduced the activation of the Akt signaling pathway and the autophagy-related protein Beclin-1, both involved in cellular survival and therapy resistance. Downregulation of these pathways contributed to decreased proliferation and increased susceptibility of resistant breast cancer cells to treatment [66].

In addition to these effects, selenium derivatives such as symmetrical selenoesters have demonstrated antitumor activity in doxorubicin-resistant breast cancer models. These compounds were shown to inhibit cancer cell proliferation and enhance the cytotoxic effects of doxorubicin, suggesting that selenium-based molecules may help overcome multidrug resistance mechanisms. The proposed mechanisms include increased oxidative stress, modulation of apoptotic pathways, and interference with cellular processes associated with drug resistance [67].

The synergistic effects observed in these studies are likely mediated through multiple mechanisms, including enhanced ROS generation, inhibition of survival signaling pathways such as PI3K/Akt, and increased activation of apoptotic pathways. Selenium compounds may also interfere with resistance mechanisms such as drug efflux pump activity and pro-survival signaling, commonly upregulated in resistant cancer cells. Consequently, combination therapies involving selenium compounds and conventional anticancer agents may allow for lower therapeutic doses while maintaining or improving treatment efficacy, thereby reducing toxicity and overcoming resistance in breast cancer [65,66,67].

## 7. Experimental Evidence in Resistant Breast Cancer Models

### 7.1. Trastuzumab-Resistant Cells

Resistance to trastuzumab represents a major clinical challenge in the treatment of HER2-positive breast cancer. Although trastuzumab has significantly improved clinical outcomes in patients with HER2-overexpressing tumors, a considerable proportion of patients develop intrinsic or acquired resistance [68,69]. Several molecular mechanisms have been proposed to explain trastuzumab resistance, including alterations in HER2 signaling, activation of compensatory growth factor pathways, and dysregulation of intracellular signaling cascades regulating cell survival and proliferation. Among these mechanisms, persistent activation of downstream signaling pathways such as the mitogen-activated protein kinase (MAPK/ERK) pathway and the PI3K/Akt pathway has been identified as a key contributor to therapeutic resistance. Trastuzumab-resistant breast cancer cell lines, including JIMT-1 cells, demonstrate sustained activation of these pro-survival pathways, enabling tumor cells to maintain proliferative signaling and evade apoptosis despite continued HER2-targeted therapy [68,69].

The PI3K/Akt pathway plays a key role in mediating resistance to anti-HER2 therapies. Activation of this pathway promotes processes that favor tumor progression, including increased cell survival, enhanced metabolic activity, and resistance to apoptotic signaling. Dysregulation of PI3K/Akt signaling can occur through several mechanisms, including activating mutations in PI3K, loss of the tumor suppressor PTEN, or increased signaling from upstream receptor tyrosine kinases. These alterations lead to persistent phosphorylation and activation of Akt, which regulates downstream targets involved in cell cycle progression, protein synthesis, and inhibition of programmed cell death. Consequently, sustained PI3K/Akt signaling is a key determinant of resistance to trastuzumab therapy in HER2-positive breast cancer models [69].

Experimental studies have demonstrated that selenium compounds, particularly sodium selenite, possess significant anticancer activity in breast cancer cells, including trastuzumab-resistant models. Sodium selenite has been shown to inhibit cell proliferation and reduce the viability of HER2-positive breast cancer cells in a dose-dependent manner. In trastuzumab-resistant JIMT-1 cells, treatment with sodium selenite significantly decreases cell viability and suppresses cellular proliferation, suggesting that selenium compounds interfere with signaling pathways that maintain tumor cell survival [66]. These findings indicate that selenium may represent a promising therapeutic agent capable of targeting signaling mechanisms involved in resistance to HER2-directed therapies.

The antiproliferative activity of sodium selenite appears to be closely associated with its ability to modulate intracellular survival signaling pathways, particularly the Akt pathway. Akt serves as a central regulator of cell survival and growth, integrating signals from multiple upstream receptors and controlling numerous downstream pathways involved in cellular metabolism, protein synthesis, and cell cycle progression. In HER2-positive breast cancer cells, Akt activation contributes to resistance against targeted therapies by promoting anti-apoptotic signaling and suppressing cellular stress responses. Inhibition of Akt signaling is therefore an important strategy for restoring therapeutic sensitivity in resistant cancer cells. Studies have demonstrated that sodium selenite treatment results in a significant reduction in phosphorylated Akt levels, thereby impairing the activity of this critical survival pathway and promoting growth inhibition in trastuzumab-resistant breast cancer cells [66].

Inhibition of Akt signaling affects downstream targets regulating proliferation and cell cycle progression. For example, suppression of PI3K/Akt activity has been shown to influence the expression and activity of proteins such as p27 and cyclin D1, which play essential roles in the control of the G1–S phase transition during the cell cycle. Reduced Akt signaling may induce cell cycle arrest and decreased proliferation in HER2-positive breast cancer cells exposed to therapeutic agents [70]. In addition, selenium compounds have been shown in various cancer models to inhibit Akt phosphorylation and disrupt survival signaling pathways, further supporting the role of selenium-mediated modulation of Akt activity in the suppression of tumor growth [65,71].

Further experimental evidence suggests that sodium selenite may enhance the efficacy of trastuzumab through combinational effects on survival signaling pathways. In vitro studies have demonstrated that sodium selenite significantly reduces cell viability in both trastuzumab-sensitive (SK-BR-3) and trastuzumab-resistant (JIMT-1) breast cancer cells. Importantly, combination treatment with sodium selenite and trastuzumab produces cytotoxic effects that are comparable to or greater than those observed with either treatment alone. In trastuzumab-resistant JIMT-1 cells, combined treatment results in decreased levels of phosphorylated Akt as well as reduced expression of Beclin-1, suggesting that sodium selenite enhances trastuzumab efficacy by inhibiting survival signaling pathways and modulating autophagy-related processes that contribute to therapeutic resistance [66].

Autophagy is a tightly regulated cellular process responsible for the degradation and recycling of intracellular components during metabolic stress or exposure to therapeutic agents. In cancer cells, autophagy may function as an adaptive survival mechanism that enables tumor cells to withstand unfavorable conditions, including treatment with anticancer drugs. Increased autophagic activity has been observed in several therapy-resistant cancers, where it contributes to maintenance of cellular homeostasis and promotes resistance to apoptosis. In trastuzumab-resistant breast cancer cells, sodium selenite treatment has been associated with decreased expression of Beclin-1, a key regulator of autophagy initiation. Suppression of autophagy-related pathways may therefore reduce the ability of cancer cells to adapt to therapeutic stress and enhance the cytotoxic effects of HER2-targeted therapies [66,72].

In addition to its effects on intracellular signaling pathways and autophagy regulation, sodium selenite may also exert anticancer activity through the induction of oxidative stress. Selenium compounds are known to influence cellular redox homeostasis by increasing the intracellular production of ROS. Elevated ROS levels can disrupt mitochondrial function, damage cellular macromolecules, and activate stress-related signaling pathways that ultimately lead to apoptotic cell death. Cancer cells often maintain a delicate balance between ROS generation and antioxidant defense mechanisms to sustain rapid proliferation while avoiding excessive oxidative damage. Disruption of this redox balance can therefore selectively impair tumor cell survival. Sodium selenite has been shown to promote ROS accumulation in cancer cells, which may contribute to mitochondrial dysfunction, activation of caspase-dependent apoptotic pathways, and inhibition of survival signaling networks. In the context of trastuzumab-resistant breast cancer, increased oxidative stress induced by selenium compounds may further weaken cellular defense mechanisms and enhance sensitivity to therapeutic interventions. Consequently, modulation of intracellular redox balance represents an additional mechanism by which selenium compounds may exert cytotoxic effects in resistant HER2-positive breast cancer models [73].

Collectively, these findings indicate that sodium selenite exerts multiple anticancer effects in trastuzumab-resistant breast cancer cells. By inhibiting the PI3K/Akt signaling pathway, suppressing autophagy-related survival mechanisms, disrupting intracellular redox balance, and influencing key regulators of cell cycle progression, sodium selenite can significantly reduce tumor cell viability and proliferation. Furthermore, the ability of sodium selenite to enhance the cytotoxic effects of trastuzumab suggests that selenium compounds may function as potential adjuvant agents capable of improving the efficacy of HER2-targeted therapies. Although these findings are primarily derived from in vitro experimental models, they provide important mechanistic insights into how selenium-based compounds may help overcome resistance to trastuzumab in HER2-positive breast cancer. Further investigation, particularly in vivo models and clinical studies, will be necessary to determine whether these experimental observations can be translated into effective therapeutic strategies for patients with trastuzumab-resistant disease [73].

### 7.2. Doxorubicin-Resistant Breast Cancer Cells

Resistance to doxorubicin is a major limitation in the treatment of breast cancer and is associated with a range of molecular mechanisms that reduce drug efficacy and promote tumor cell survival. One of the most prominent mechanisms involves the overexpression of ATP-binding cassette (ABC) transporters, particularly ABCB1 (P-glycoprotein), which actively exports chemotherapeutic agents from cancer cells, reducing intracellular drug accumulation and limiting cytotoxic activity. Breast cancer cell lines characterized by high ABCB1 expression are widely used as experimental models to investigate strategies for overcoming multidrug resistance (MDR) [74].

In addition to enhanced drug efflux, doxorubicin resistance is also associated with alterations in intracellular signaling pathways that regulate cell survival and apoptosis. Dysregulation of pathways such as PI3K/Akt increases resistance by promoting anti-apoptotic signaling and metabolic adaptation. Moreover, changes in the activity of topoisomerase II, the primary molecular target of doxorubicin, may further reduce the effectiveness of DNA damage-mediated cytotoxicity. These adaptations enable cancer cells to survive despite exposure to chemotherapeutic stress [75].

Experimental evidence indicates that selenium-containing compounds, particularly selenoesters, exhibit potent anticancer activity in doxorubicin-resistant breast cancer cells, including cells with high ABCB1 expression. Selenoesters reduce cell viability and induce apoptosis in resistant cell populations. The compounds induce apoptosis via caspase-dependent pathways, disruption of mitochondrial membrane potential, and modulation of the balance between pro-apoptotic and anti-apoptotic proteins, ultimately leading to programmed cell death even in cells resistant to conventional chemotherapy [67].

Importantly, selenoesters have been reported to restore sensitivity to doxorubicin in resistant cell lines. This resensitization is associated with increased intracellular accumulation of the chemotherapeutic agent, suggesting that selenoesters interfere with the function or expression of ABCB1 transporters. By impairing drug efflux, they enhance intracellular drug retention and restore the cytotoxic potential of doxorubicin [67].

Further evidence highlights the role of selenium-based nanomaterials in overcoming doxorubicin resistance. Studies using breast cancer cell lines such as MCF-7 and MDA-MB-231, as well as additional models including HCT116 and Caco-2, have demonstrated that selenium nanoparticles (nano-Se) combined with doxorubicin significantly enhances cytotoxicity, increases apoptosis, and reverses the MDR phenotype. These effects are associated with modulation of redox balance and increased levels of trace elements such as zinc, which may further influence cellular stress responses and apoptotic signaling pathways [75].

In addition to their effects on drug transport and apoptosis, selenium compounds contribute to the disruption of redox homeostasis in resistant cancer cells. By increasing intracellular reactive oxygen species (ROS) levels beyond the buffering capacity of antioxidant systems, selenoesters and selenium nanoparticles induce oxidative stress, mitochondrial dysfunction, and activation of stress-related signaling pathways. Since doxorubicin-resistant cells often rely on enhanced antioxidant defenses, this disruption of redox balance represents a particularly effective strategy for promoting cell death and overcoming resistance [67].

Taken together, these findings demonstrate that selenium compounds target multiple interconnected mechanisms involved in doxorubicin resistance, including drug efflux, survival signaling, apoptotic regulation, and redox homeostasis. Through these combined effects, selenium-based agents enhance the sensitivity of resistant breast cancer cells to doxorubicin and suppress tumor cell proliferation in experimental models.

### 7.3. Tamoxifen—Breast-Resistant Cells

Resistance to tamoxifen represents one of the major clinical challenges in the treatment of estrogen receptor-positive (ER+) breast cancer. Although tamoxifen has been widely used for decades as an effective selective estrogen receptor modulator (SERM), a substantial proportion of patients eventually develop either intrinsic or acquired resistance to this therapy [76]. At the molecular level, tamoxifen resistance is frequently associated with alterations in estrogen receptor signaling, including changes in ER expression, receptor conformation, co-regulator recruitment, and transcriptional activity. In addition, resistant cells often activate alternative proliferative and survival pathways that bypass ER signaling. Among the most frequently implicated pathways are the PI3K/Akt and MAPK signaling pathways, both of which can promote cell proliferation, survival, and resistance to endocrine therapy. Activation of these pathways may result from mutations, receptor tyrosine kinase signaling, or crosstalk between growth factor receptors and ER networks. Consequently, cancer cells can maintain proliferative capacity and survival even in the presence of continued anti-estrogen treatment. Understanding the molecular mechanisms underlying endocrine resistance is therefore essential for identifying new therapeutic strategies capable of restoring treatment sensitivity and improving patient outcomes [76].

In recent years, increasing attention has been directed toward the potential anticancer properties of selenium compounds, particularly MSA, a biologically active metabolite of selenium. Experimental studies have demonstrated that MSA exerts antiproliferative and pro-apoptotic effects in breast cancer cells. One of the key mechanisms responsible for these effects involves the modulation of estrogen receptor signaling. MSA influences the expression and functional activity of ERα, resulting in reduced receptor levels and estrogen-dependent transcriptional activity. Through this mechanism, MSA interferes with the ER-responsive gene regulation controlling cell cycle progression, proliferation, and cell survival. By disrupting estrogen-dependent signaling pathways, MSA may limit the ability of breast cancer cells to maintain growth under endocrine therapy conditions, thereby contributing to the inhibition of tumor cell proliferation [77]. Beyond estrogen receptor signaling, MSA can inhibit the growth of tamoxifen-resistant breast cancer cells through several complementary molecular mechanisms. One important effect involves the induction of apoptosis through activation of caspase-dependent pathways. Increased activation of caspase-3 and other downstream apoptotic mediators has been observed after MSA treatment, indicating that selenium compounds can promote programmed cell death in breast cancer cells. Additionally, MSA has been reported to influence the expression and activity of cell cycle-regulatory proteins, leading to cell cycle arrest and suppression of uncontrolled proliferation. Another relevant mechanism involves the modulation of cellular redox balance. Selenium compounds can alter intracellular ROS levels and oxidative stress responses, contributing to mitochondrial dysfunction, DNA damage, and activation of apoptotic pathways. These combined mechanisms contribute to the overall growth-inhibitory effects observed in breast cancer cells exposed to MSA [78].

Importantly, several studies suggest that selenium compounds may also enhance the sensitivity of breast cancer cells to endocrine therapy. By altering estrogen receptor expression and interfering with ER-mediated transcription, MSA can partially restore regulatory control over hormone-dependent signaling pathways that are frequently dysregulated in resistant cells. Furthermore, inhibition of alternative survival pathways and induction of apoptosis may enhance tamoxifen’s antiproliferative effects in combination. This potential synergistic interaction between tamoxifen and selenium compounds suggests that MSA could function as a therapeutic adjuvant capable of improving treatment efficacy in tamoxifen-resistant breast cancer models. Such findings highlight the possibility that selenium-based compounds may contribute to overcoming endocrine resistance and improving therapeutic responses in patients with hormone-dependent breast cancer [77,78,79].

An additional layer of complexity in tamoxifen resistance involves the dynamic interplay between estrogen receptor signaling, growth factor pathways, and cellular stress responses. Increasing evidence suggests that persistent activation of PI3K/Akt and MAPK pathways may lead to phosphorylation of ERα, resulting in ligand-independent receptor activation and sustained transcription of proliferative genes even in the absence of estrogen. This phenomenon further diminishes the antagonistic effects of tamoxifen and promotes a resistant phenotype. Moreover, these signaling pathways are closely linked to cellular redox regulation, as Akt-mediated signaling can enhance antioxidant defenses and support cancer cell survival under oxidative stress. In this context, selenium compounds such as MSA exert a dual effect by disrupting ER signaling and adaptive redox responses. By increasing intracellular ROS levels while inhibiting survival signaling, MSA can shift the cellular balance toward apoptosis and reduce the threshold required for cell death induction. This integrated targeting of receptor signaling, survival pathways, and oxidative stress responses represents a particularly effective strategy for overcoming endocrine resistance at the molecular level [76,77,78,79].

Overall, current experimental evidence indicates that methylseleninic acid can modulate estrogen receptor signaling, disrupt proliferative pathways, and promote apoptotic cell death in breast cancer cells. These mechanisms collectively contribute to significant inhibition of tumor cell growth and may enhance the responsiveness of resistant cells to endocrine therapy. Although most available data originate from in vitro and preclinical studies, these findings support the potential role of selenium compounds as adjunctive therapeutic agents in the management of hormone-resistant breast cancer. Further investigation, particularly in clinical settings, is required to determine whether these experimental observations can be translated into effective therapeutic strategies for patients with tamoxifen-resistant disease [76,77,78,79].

### 7.4. Paclitaxel—Breast Cancer-Resistant Cells

Taxanes, such as paclitaxel, are widely used chemotherapeutic agents in breast cancer. Resistance to paclitaxel represents a major obstacle to effective breast cancer treatment. Paclitaxel stabilizes microtubules, disrupting mitosis and inducing apoptosis [80]. Despite this, resistant breast cancer cells develop multiple adaptive mechanisms that allow them to evade these cytotoxic effects. These mechanisms include altered redox homeostasis, activation of pro-survival signaling pathways such as Akt and NF-κB, and enhanced cellular defense systems that reduce susceptibility to apoptosis. Consequently, there is considerable interest in identifying agents capable of overcoming taxane resistance [74,80].

Recent experimental studies have shown that selenium compounds, such as selenite and synthetic derivatives (such as selenoesters), exhibit significant cytotoxic activity in paclitaxel-resistant breast cancer cells. In addition, these compounds induce apoptosis in cancer cells that are resistant to paclitaxel treatment. These effects are associated with increased production of ROS, leading to oxidative stress, mitochondrial dysfunction and activation of caspase-dependent cell death pathways. Moreover, selenium has been shown to inhibit important survival signaling pathways, including Akt and NF-κB, often upregulated in resistant cells and contribute to continued proliferation and resistance to apoptosis. Inhibition of these pathways further enhances the susceptibility of resistant cells to cytotoxic stimuli [82,83].

Another important experimental study has demonstrated that K1–K7, ketone-containing selenoesters, showed stronger cytotoxic effects than cyanoselenoesters. These compounds, particularly K7, show strong anticancer activity and sensitize drug-resistant cancer cells to chemotherapy by inhibiting ABC efflux pump. Notably, K7 significantly enhanced the effectiveness of adriamycin and paclitaxel in resistant cells [83,84].

An additional mechanism contributing to paclitaxel resistance involves alterations in microtubule composition and mitotic checkpoint regulation. Overexpression of specific β-tubulin isotypes, particularly βIII-tubulin, can reduce paclitaxel binding affinity and alter microtubule dynamics, thereby diminishing drug-induced mitotic arrest. Furthermore, dysregulation of the spindle assembly checkpoint enables cancer cells to bypass mitotic arrest and continue proliferation despite microtubule stabilization. These structural and regulatory adaptations are often accompanied by activation of survival signaling pathways such as PI3K/Akt and NF-κB, which further suppress apoptosis and promote resistance. Importantly, these pathways are linked to cellular redox balance, as enhanced antioxidant capacity allows resistant cells to tolerate increased oxidative stress. Selenium compounds may counteract these mechanisms by simultaneously disrupting microtubule-associated survival signaling, inhibiting Akt and NF-κB activation, and increasing intracellular ROS levels. This coordinated disruption of cytoskeletal dynamics, survival pathways, and redox homeostasis lowers the threshold for apoptosis induction and enhances the cytotoxic response of paclitaxel-resistant breast cancer cells [80,81,82,83,84].

Functionalized nano-Se has also been explored for selective transport of paclitaxel to tumor cells, where nano-Se improves therapeutic efficacy by promoting ROS-mediated cytotoxicity and altering cancer cell signaling. It has been suggested that nano-Se may have potential synergistic effects when selenium is combined with conventional taxanes [84].

### 7.5. Toxicity and Dose

Although selenium compounds are generally associated with relatively low toxicity compared to conventional chemotherapeutic agents, their biological effects are highly dose-dependent, and the therapeutic window is relatively narrow. Selenium is an essential micronutrient required at low doses, with a recommended dietary intake of approximately 55 µg/day in adults. However, excessive intake can lead to toxicity, and the tolerable upper intake level is established at 400 µg/day for adults [85]. At concentrations above physiological requirements, selenium compounds can induce adverse effects collectively referred to as selenosis.

Selenosis is associated with a range of clinical manifestations, including gastrointestinal disturbances, fatigue, irritability, hair and nail brittleness or loss, and a characteristic garlic-like odor of the breath due to volatile selenium metabolites. In more severe cases, neurological abnormalities and organ dysfunction may occur. Chronic exposure to elevated selenium levels has been linked to disturbances in redox homeostasis and cellular toxicity, reflecting the dual role of selenium as both an antioxidant and a pro-oxidant depending on its concentration [6].

Importantly, experimental studies demonstrating anticancer effects of selenium compounds often utilize concentrations that exceed physiological levels, raising concerns regarding their translational applicability. High-dose selenium supplementation (e.g., 1600–3200 µg/day) has been associated with increased incidence of toxicity-related symptoms, even in the absence of severe organ damage. Furthermore, the therapeutic effects of selenium appear to follow a U-shaped dose–response relationship, where both deficiency and excess may have detrimental biological consequences. Taken together, these findings indicate that although selenium compounds exhibit promising anticancer activity in preclinical models, their safety profile requires careful consideration. The narrow margin between beneficial and toxic doses highlights the need for precise dose optimization and underscores the importance of evaluating selenium speciation, baseline selenium status, and long-term toxicity in future clinical studies [91,92].

## 8. Conclusions

Selenium compounds exhibit significant anticancer potential by modulating redox homeostasis, inducing apoptosis, and regulating key signaling pathways involved in tumor progression and therapy resistance. Their diverse chemical forms, including organic selenium compounds and selenium nanoparticles, offer promising opportunities for novel anticancer strategies. However, despite encouraging preclinical findings, several critical challenges remain.

First, the dose-dependent and context-specific effects of selenium, often described as a U-shaped relationship, complicate therapeutic application and require careful optimization. In addition, inconsistent clinical outcomes and limited clinical trial data highlight the need for well-designed studies to evaluate the efficacy and safety of selenium-based therapies across different cancer types and patient populations [88].

Furthermore, although selenium nanoparticles demonstrate improved selectivity and reduced toxicity, their pharmacokinetics, long-term safety and targeted delivery mechanisms remain insufficiently characterized, limiting their translational potential [89,90]. Mechanistic understanding also remains incomplete, particularly regarding the role of selenium in epigenetic regulation, non-coding RNAs and tumor microenvironment interactions, which warrants further investigation [89,90].

Despite extensive preclinical research published between 2005 and 2026 and identified through PubMed, Scopus, and Web of Science databases, the translational potential of selenium compounds remains only partially realized. Most of the available evidence is derived from in vitro studies, with limited in vivo validation and a marked lack of clinical evidence, limiting the ability to draw firm conclusions regarding therapeutic efficacy in patients. Importantly, the current evidence base is also limited by methodological weaknesses in many key studies, including small sample sizes, variability in experimental design, and insufficient or inconsistent reporting of effect sizes. In several cases, lack of standardized controls and differences in outcome measures reduce the reproducibility and comparability of results. In addition, potential publication bias toward positive findings further increases the risk of overestimating the observed anticancer effects. Taken together, selenium compounds remain a biologically active and extensively studied class of agents; however, their clinical application in breast cancer therapy is still insufficiently developed. Future research should focus on well-designed, standardized preclinical and clinical studies, including robust assessment of effect sizes and methodological quality, as well as subtype-specific responses in breast cancer, to better define their therapeutic potential and facilitate their possible integration into clinical oncology. A deeper understanding of selenium’s molecular mechanisms, combined with advances in nanotechnology and precision medicine, may ultimately enable its more effective and evidence-based integration into cancer therapy [1,2,3,4,5,6,7,8,9,10,11,12,13,14,15,16,17,18,19,20,21,22,23,24,25,26,27,28,29,30,31,32,33,34,35,36,37,38,39,40,41,42,43,44,45,46,47,48,49,50,51,52,53,54,55,56,57,58,59,60,61,62,63,64,65,66,67,68,69,70,71,72,73,74,75,76,77,78,79,80,81,82,83,84,85,86,87,88,89,90].

## Figures and Tables

**Table 1:** Selenoproteins and their biological functions.

Selenoprotein	General Biological Functions	Ref
GPX (Glutathione Peroxidases)	Antioxidants reduce hydrogen and lipid peroxides. They are important in the cell’s protection against oxidative stress.	[7]
TXNRD (Thioredoxin Reductases)	The regulation of redox homeostasis, keeping cellular redox balance in signaling pathways.	[7]
DIO (Iodothyronine Deiodinases: DIO1, DIO2, DIO3)	The conversion of thyroid hormones (T4 → T3); regulation of body metabolism.	[7]
SELENOP (Selenoprotein P)	The transport of selenium in the bloodstream; antioxidant protection within the serum.	[11]
SELENOW (Selenoprotein W)	Regulation of redox homeostasis; neuroprotective function.	[11]
SELENOS (Selenoprotein S)	The regulation of inflammation processes; regulation of immunological response; ER-associated degradation.	[6]
SELENOK (Selenoprotein K)	They support immune systems, regulate the amount of calcium in cells, and influence lymphocyte proliferation.	[13]
SELENOM (Selenoprotein M)	Neuroprotection function.	[14]
SELENON (Selenoprotein N)	The regulation of muscle function; protection against the endoplasmic reticulum stress.	[15]
GPX4 (Glutathione Peroxidase 4)	The protection against lipid peroxidation and ferroptosis.	[16]
SELENOH	Regulation of redox homeostasis and genome stability; suppression of cellular senescence.	[17]
SELENOT	Endoplasmic reticulum-resident oxidoreductase; maintenance of ER homeostasis and protection against oxidative stress.	[18]
SELENOF (Selenoprotein F)	Endoplasmic reticulum-resident thiol-disulfide oxidoreductase involved in protein folding quality control and redox regulation.	[86]
SELENOI (Selenoprotein I)	Ethanolamine phosphotransferase involved in phospholipid biosynthesis; essential for membrane formation and cellular metabolism.	[87]

## Data Availability

No new data were created or analyzed in this study. Data sharing is not applicable to this article.

## Abbreviations

SECIS	Selenocysteine Insertion Sequence
ROS	Reactive Oxygen Species
GPX	Glutathione Peroxidases
GPX4	Glutathione Peroxidase 4
TXNRD/TrxRs	Thioredoxin Reductases
DIO	Iodothyronine Deiodinases
DIO1	Type 1 Iodothyronine Deiodinase
DIO2	Type 2 Iodothyronine Deiodinase
DIO3	Type 3 Iodothyronine Deiodinase
SELENOP/SelP	Selenoprotein P
SELENOW	Selenoprotein W
SELENOS/SelS	Selenoprotein S
SELENOK	Selenoprotein K
SELENOM/SelM	Selenoprotein M
SELENON	Selenoprotein N
SelF	Selenoprotein F
SeNPs	Selenium Nanoparticles
NKG2D	Natural Killer Group 2 Member D
ULBP2	UL16 Binding Protein 2
MSA	Methylseleninic Acid
BCL-2	B-cell Lymphoma 2
BAX	BCL-2 Associated X Protein
NO	Nitric Oxide
MDA	Malondialdehyde
GSH	Glutathione
SOD	Superoxide Dismutase
MAPK	Mitogen-Activated Protein Kinase
MAPK/ERK	Mitogen-Activated Protein Kinase/Extracellular Signal-Regulated Kinase
SK-BR-3	Trastuzumab-Sensitive Breast Cancer Cell Line
JIMT-1	Trastuzumab-Resistant Breast Cancer Cell Line
